# Thiotepa-busulfan-fludarabine compared to busulfan-fludarabine for sibling and unrelated donor transplant in acute myeloid leukemia in first remission

**DOI:** 10.18632/oncotarget.23273

**Published:** 2017-12-15

**Authors:** Francesco Saraceni, Myriam Labopin, Rose-Marie Hamladji, Ghulam Mufti, Gerard Socié, Avichai Shimoni, Jeremy Delage, Eric Deconinck, Patrice Chevallier, Didier Blaise, Jaime Sanz, Anne Huynh, Edouard Forcade, Bipin N. Savani, Mohamad Mohty, Arnon Nagler, Acute Leukemia Working Party of the European society for Blood and Marrow Transplantation (EBMT)

**Affiliations:** ^1^ Hematology and Stem Cell Transplant, Ravenna Hospital, Ravenna, Italy; ^2^ EBMT Paris Study Office, Saint Antoine Hospital, Paris, France; ^3^ Centre Pierre et Marie Curie, Service Hématologie Greffe de Moëlle, Alger, Algeria; ^4^ GKT School of Medicine, Department of Haematological Medicine, London, United Kingdom; ^5^ Hopital St. Louis, Department of Hematology-BMT, Paris, France; ^6^ Chaim Sheba Medical Center, Department of Bone Marrow Transplantation, Tel-Hashomer, Israel; ^7^ CHU Lapeyronie, Département d’Hématologie Clinique, Montpellier, France; ^8^ Hopital Jean Minjoz, Service d’Hématologie, Besançon, France; ^9^ CHU Nantes, Department D’Hématologie, Nantes, France; ^10^ Programme de Transplantation and Therapie Cellulaire, Centre de Recherche en Cancérologie de Marseille, Institut Paoli Calmettes, Marseille, France; ^11^ Hospital Universitario La Fe, Servicio de Hematologia, Valencia, Spain; ^12^ Institut Universitaire du Cancer Toulouse, Oncopole, I.U.C.T-O, Toulouse, France; ^13^ CHU Bordeaux, Hôpital Haut-Leveque, Pessac, France; ^14^ Vanderbilt University Medical Center, Nashville, TN, USA; ^15^ Department of Haematology, Saint Antoine Hospital, Paris, France

**Keywords:** acute myeloid leukemia (AML), allogeneic transplantation, thiotepa-busulfan-fludarabine (TBF), busulfan-fludarabine (BF), myeloablative conditioning (MAC)

## Abstract

**Background:**

A preparatory regimen consisting of thiotepa-busulfan-fludarabine (TBF) has been associated with reduced relapse in patients with haematological malignancies after haploidentical and cord blood transplants; however, few data exist regarding TBF conditioning in sibling (MSD) and unrelated donor (URD) transplants for AML.

**Results:**

Among patients receiving a myeloablative (MAC) regimen, TBF-MAC was associated with significantly lower relapse (HR 0.47, *p* = 0.005) however higher non-relapse mortality (NRM, HR 2.69, *p* < 10^–4^) as compared to BF. This led to similar leukemia-free (LFS) and overall survival (OS) between the two regimens (LFS: *p* = 0.6; OS: *p* = 0.27). When we selected TBF-MAC patients receiving busulfan 9.6 mg/kg, NRM resulted still higher but no more significantly different as compared to BF-MAC with busulfan 12.8 mg/kg (HR 1.53, *p* = 0.12); despite the lower busulfan dose, relapse remained inferior with TBF-MAC (HR 0.45, *p* = 0.01), however no difference in survival could be demonstrated (LFS: *p* = 0.31; OS: 0.82). Among patients receiving a reduced-intensity (RIC) regimen, similar outcome was observed with TBF-RIC and BF-RIC (LFS: *p* = 0.77; OS: *p* = 0.88).

**Conclusions:**

TBF-MAC as conditioning regimen for transplant from MSD and URD in AML patients in first remission provided stronger anti-leukemic activity but higher NRM as compared to BF-MAC, thus leading to similar survival. TBF-MAC with busulfan 9.6 mg/kg was associated with low relapse and acceptable NRM, however again with no survival benefit. TBF-RIC and BF-RIC resulted in comparable outcome.

**Methods:**

We conducted a registry-based study comparing outcomes of patients with AML in first remission undergoing transplant from MSD or URD prepared with either TBF (*n* = 212) or BF (*n* = 2698) conditioning.

## INTRODUCTION

In recent years, the paradigm of conditioning regimens for allogeneic stem cell transplant (allo-SCT) in AML has shifted from heavy myeloablation to reduced intensity protocols, with the aim of minimizing regimen related toxicity while exploiting the graft-versus-leukemia effect [1]. An important step towards this goal has been made with the replacement of cyclophosphamide with fludarabine [2]. A preparatory regimen consisting of busulfan and fludarabine (BF) was initially described in the early 2000s; [3] subsequently different studies were carried out to challenge BF with standard BuCy, reporting conflicting results on the relative merits of the two regimens in terms of non-relapse mortality (NRM), relapse and survival [4–7]. More recently, different prospective randomized trials have been conducted in an attempt to answer the question whether BF could be a valid substitution for standard BuCy, reporting inconsistent results [8–11]. A recent meta-analysis of 15 studies [12] concluded that the BF conditioning regimen is associated with inferior toxicity and lower 100-days NRM with no increase in relapse risk as compared to BuCy; however, no survival benefit could be demonstrated. In fact, although the substitution of cyclophosphamide with fludarabine results in decreased toxicity, leukemia relapse still represents the main cause of transplant failure, accounting for about 30% at 2 years when transplant is performed in remission.

Thiotepa is an alkylating compound with immunosuppressive properties which holds favourable characteristics as the capability of penetrating the blood-brain barrier combined with a reduced non-haematologic toxicity, which led to a widespread use of this compound within transplant preparative regimens [13–15]. A conditioning protocol consisting of thiotepa, busulfan and fludarabine (TBF), initially designed for cord blood transplant, [16, 17] has recently been associated with low relapse rates and satisfactory outcome in haploidentical transplantation (haplo-SCT) [18–22] and in single center reports of allo-SCT from MSD and URD-SCT for different haematological malignancies [23, 24]. However, no data are available about outcome of AML patients undergoing MSD or URD-SCT prepared with this protocol, and no comparison of this novel regimen with standard regimens has been published yet. We therefore took advantage of the EBMT dataset, and retrospectively compared outcome following TBF versus BF as conditioning regimens before allo-SCT from MSD or URD-SCT in a large homogeneous population of AML patients undergoing transplant in first complete remission (CR1).

## RESULTS

### Patient characteristics

Patients from 204 transplant centers were included. The total number of patients who received either TBF or BF in CR1 between January 2007 and June 2015 and reported to the EBMT was 4995 (265 TBF and 4730 BF); two thousand and eighty-five patients were excluded from the analysis as they have received a busulfan dose <6.4 mg/kg, thus leading to a final number of 2910 patients; among them, 212 received TBF and 2698 BF, respectively. One thousand six hundred and six patients received a MAC and 1304 a RIC regimen. One thousand six hundred and fifty-three (57%) patients were transplanted from MSD, 987 (34%) from 10/10 URD and 9% (270) from 9/10 URD, respectively**.** Data about cytogenetic risk were available for 55% of patients, while information on molecular genetics and minimal residual disease (MRD) status was missing for most patients and was therefore not included in the analysis. The characteristics of patients are summarized in Table 1.

**Table 1:** Patient, disease and transplant characteristics

		Type of transplant				*P*	
		MAC		RIC		MAC	RIC
		TBF-MAC	BF-MAC	TBF-RIC	BF-RIC		
**Number (total: 2910)**		*147*	*1459*	65	1239		
**Follow up, months (range)**		15 (1–75)	19 (1–111)	19 (1-111)	15 (1–75)		
**Age at transplant, years (range; IQR)**		45 (19–66; 34–52)	50 (18–74; 39–57)	60 (27–72; 56–63)	60 (19–75; 54–64)	<10^–3^	0.79
**Median year of transplant (range)**		2014 (2008–2015)	2013 (2007–2015)	2014 (2008–2015)	2012 (2007–2015)	<10^–3^	<10^–3^
**Cytogenetic risk, *n* (%)**	Favorable	9 (16%)	65 (11%)	1 (3%)	53 (6%)	0.285	0.093
	Intermediate-I	7 (13%)	122 (20%)	6 (21%)	250 (28%)		
	Intermediate-II	16 (29%)	125 (20%)	3 (10%)	160 (18%)		
	Adverse	6 (11% )	88 (14%)	2 (7%)	128 (14% )		
	missing	91	848	36	348		
**Diagnosis, *n* (%)**	De novo AML	129 (88%)	1248 (86%)	48 (74%)	939 (76%)	0.464	0.722
	Secondary AML	18 (12%)	211 (14%)	17 (26%)	300 (24%)		
**Karnofsky PS, *n* (%)**	<80%	2 (1% )	19 (1% )	1 (2% )	59 (5.% )	0.985	0.227
	≥80%	145 (99% )	1358 (99% )	59 (98% )	1088 (94% )		
	<90%	17 (12% )	207 (15% )	12 (20% )	291 (25% )	0.259	0.342
	≥90%	130 (88% )	1170 (85% )	48 (80% )	852 (74% )		
**Donor, *n* (%)**	MSD	75 (51%)	944 (65%)	39 (60%)	595 (48%)	0.001	0.113
	10/10 URD	50 (34%)	396 (27%)	23 (35%)	518 (42%)		
	9/10 URD	22 (15%)	119 (8%)	3 (5%)	126 (10%)		
**Stem cell source, *n* (%)**	BM	50 (34%)	247 (17%)	11 (17%)	62 (5%)	<10^–3^	<10^–3^
	PBSC	97 (66%)	1212 (83%)	54 (83%)	1177 (95%)		
**Patient gender, *n* (%)**	male	74 (50%)	787 (54%)	38 (59%)	657 (53%)	0.38	0.327
	female	73 (50%)	667 (46%)	26 (41%)	580 (47%)		
**Donor gender, *n* (%)**	Male	102 (69%)	858 (59%)	35 (55%)	772 (63%)	0.018	0.179
	Female	45 (31%)	587 (41%)	29 (45%)	453 (37%)		
	Missing	0	14	1	14		
**Donor/recipient gender matching, *n* (%)**	No F to M	122 (83%)	1126 (78%)	48 (75%)	1010 (83%)	0.176	0.122
	F to M	25 (17%)	314 (22%)	16 (25%)	213 (17%)		
	Missing	0	19	1	16		
**Patient CMV serology, *n* (%)**	negative	31 (22%)	324 (23%)	8 (13%)	417 (34% )	0.806	<10^–4^
	positive	112 (78%)	1111 (77%)	55 (87%)	808 (66% )		
	Missing	4	24	2	14		
**Donor CMV serology, *n* (%)**	negative	56 (39% )	485 (34% )	18 (29% )	580 (47%)	0.229	0.003
	positive	88 (61% )	946 (66%)	45 (71% )	643 (53%)		
	Missing	3	28	2	16		
**CMV donor/recipient matching**	D–/R–	18 (13%)	215 (15%)	3 (5%)	300 (25%)	0.297	
	D+/R–	12 (9%)	107 (8%)	5 (8%)	117 (10%)		
	D–/R+	35 (25%)	264 (19%)	15 (24%)	278 (23%)		
	D+/R+	76 (54%)	830 (59%)	39 (63%)	519 (43%)		
	Missing	6	43	3	25		
***in vivo* TCD, *n* (%)**	No	67 (46%)	646 (45%)	39 (60%)	213 (17% )	0.718	<10^–4^
	Yes	78 (54%)	801 (55% )	26 (40% )	1024 (83% )		
	Missing	2	12	0	2		
**GVHD prophylaxis**	CSA + ATG	9 (6.3%)	175 (12.4%)	6 (9.2%)	391 (32.1%)	0.002	<0.001
	CSA + MTX	50 (34.5%)	527 (37.2%)	21 (32.3%)	139 (11.4%)		
	CSA + MTX + ATG	62 (43.1%)	386 (27.2%)	16 (24.6%)	208 (17.1%)		
	CSA + MMF	6 (4.2%)	60 (4.2%)	3 (4.6%)	34 (2.8%)		
	CSA + MMF + ATG	6 (4.2%)	110 (7.8%)	3 (4.6%)	319 (26.2%)		
	other	11 (7.6%)	159 (11.2%)	16 (24.6%)	127 (10.4%)		
**Busulfan dose, *n* (%)**	9.6 mg/Kg	111 (76%)	339 (23%)	111 (76%)	339 (23%)	<10^–3^	<10^–3^
	12.8 mg/Kg	36 (24%)	1120 (77%)	36 (24%)	1120 (77%)		

Data are median (IQR), median (range), median (range; IQR), *n* (%), or n/N (%). Some percentages do not add up to 100% because of rounding.

Abbreviations: BF, busulfan-fludarabine; BM, Bone marrow; CMV, Cytomegalovirus; MAC, myeloablative conditioning; MSD, matched sibling donor; URD, unrelated donor; PBSCs, peripheral blood stem cells; RIC, reduced-intensity conditioning; TBF, thiotepa-busulfan-fludarabine; TCD, T-cell depletion.

### Myeloablative conditioning: TBF-MAC versus BF-MAC

#### Patient, disease and transplant characteristics

One-hundred and forty-seven patients received TBF-MAC, while 1459 patients received the BF-MAC regimen. TBF-MAC group included significantly younger patients (median age 45 vs 50 years, *p* < 10^−3^) transplanted more recently (median year of transplant 2014 vs 2013, *p* < 10^−3^) as compared to for BF-MAC, respectively. Patients conditioned with TBF-MAC were more likely to have received a URD transplant (49% vs 35%, *p* = 0.001), BM as stem cell source (34% vs 17%, *p* < 10^−3^), and the donor most likely to be male (69% vs 59%, *p* = 0.018), as compared to BF-MAC. Cytogenetic risk, proportion of patients with secondary AML, Karnofsky performance score, proportion of patients who received *in-vivo* T-cell depletion, kind of donor and patient CMV serology did not differ between the groups. Among the TBF-MAC cohort, 111 patients (76%) received busulfan 9.6 mg/kg, while 36 (24%) received 12.8 mg/kg. Within the BF-MAC cohort, 339 patients (23%) received busulfan 9.6 mg/kg and 1120 (77%) 12.8 mg/kg.

### Engraftment, NRM and GVHD

Engraftment rate was 98% following both regimens (*p* = 0.88). The median time to neutrophil engraftment was 18 (10–47) days and 15 (5–45) days for TBF-MAC and BF-MAC, respectively (*p* < 10^−5^). The 2-year non-relapse mortality rate in the overall population was significantly higher after TBF-MAC compared to BF-MAC in both univariate (27 ± 8% vs 16 ± 2%, *p* = 0.006) and multivariate analysis (HR 2.7, *p* < 10^−4^). When analyzing separately NRM within and after day 100, NRM rate following TBF-MAC was significantly higher as compared to BF-MAC within 100 days after transplant (8 ± 4% vs 5 ± 1%, *p* = 0.008), while a trend for higher NRM was observed for the time period between 100 days and 2 years after transplant (19 ± 8% vs 12 ± 2% for TBF-MAC and BF-MAC respectively, *p* = 0.08). Karnofsky performance score at transplant (≥90% vs <90%) did not affect risk of NRM. Independent predictive factors for higher NRM risk were older age, higher busulfan dose, URD transplant compared to MSD and PBSC compared to BM graft.

Main causes of NRM were GVHD and infectious complications (Table 2). When analyzing separately the incidence of infectious-related and GVHD-related deaths no difference was observed between the two study cohorts.

**Table 2A:** Causes of death

	MAC		RIC	
	TBF	BF	TBF	BF
Total	43	501	18	468
GVHD	13 (32%)	100 (21%)	4 (24%)	100 (22%)
Infection	10 (24%)	89 (19%)	3 (18%)	78 (17%)
Original disease	12 (29%)	247 (52%)	8 (47%)	234 (52%)
VOD	2 (5%)	8 (2%)	0	0
Failure/Rejection	0	2 (1%)	0	2 (1%)
Hemorrhage	1 (2%)	1 (1%)	0	3 (1%)
Cardiac toxicity	0	1 (1%)	1 (6%)	1 (1%)
Interstitial Pneumonitis	0	6 (2%)	1 (6%)	3 (1%)
Second malignancy	1 (2%)	3 (1%)	0	6 (2%)
Other transplantation related	2 (5%)	21 (4%)	0	19 (4%)
Missing	2	23	1	22

**Table 2B:** Causes of death by time period after transplant

		MAC		RIC	
		TBF	BF	TBF	BF
**Before day 100**	Total	14	184	8	157
	Infection	4 (29%)	39 (21%)	3 (38%)	20 (13%)
	GVHD	4 (29%)	31 (17%)	0	24 (15%)
	Original disease	2 (14%)	89 (49%)	3 (38%)	100 (65%)
	Cardiac toxicity	0	0	1 (13%)	1 (1%)
	Haemorhage	1 (7%)	1 (1%)	0	1 (1%)
	Failure/Rejection	0	2 (1.1%)	0	0
	VOD	2 (14%)	8 (4%)	0	0
	Interstitial Pneumonitis	0	2	1 (13%)	1 (1%)
	Second malignancy	0	0	0	0
	Other transplantation related	1 (7%)	10 (5%)	0	8 (5%)
	Missing	0	2	0	2
**From day 100 to 2 years**	Total	25	274	8	259
	Original disease	9 (38%)	141 (54%)	4 (57%)	112 (46%)
	GVHD	8 (33%)	59 (23%)	3 (43%)	71 (29%)
	Infection	5 (21%)	44 (17%)	0	44 (18%)
	Haemorhage	0	0	0	2 (1%)
	Failure/Rejection	0	0	0	2 (1%)
	VOD	0	0	0	0
	Cardiac toxicity	0	1 (1%)	0	0
	Interstitial Pneumonitis	0	4 (2% )	0	2 (1% )
	Second malignancy	1 (4% )	1 (1% )	0	3 (1% )
	Other transplantation related	1 (4% )	10 (4% )	0	8 (3% )
	Missing	1	14	1	15

Abbreviations: BF, busulfan-fludarabine; GVHD, graft-versus-host disease; MAC, myeloablative conditioning; RIC, reduced-intensity conditioning; TBF, thiotepa-busulfan-fludarabine; VOD, veno-occlusive disease.

Veno-occlusive disease (VOD) accounted for 2 (5%) of 43 lethal complications in the TBF and 8 (2%) of 478 deaths in the BF group, respectively.

The incidence of grade II–IV and III–IV acute graft-versus-host disease (aGVHD) was similar between the groups, being 25 ± 7% and 10 ± 5% in the TBF-MAC and 24 ± 2% and 7 ± 1% in BF-MAC groups, (*p =* 0.83 and *p =* 0.22, respectively). The 2-year cumulative incidence of cGVHD of any grade was similar following TBF-MAC (35 ± 9%) compared to BF-MAC (40 ± 3%, *p =* 0.5). Similarly, no difference was observed as for the incidence of severe cGVHD (17% in both groups, *p =* 0.78). Multivariate analysis confirmed those results (Table 3).

**Table 3:** Multivariate analysis of transplantation outcome

		MAC			RIC		
Outcome		HR	95% CI	*P*	HR	95% CI	*P*
Relapse	TBF vs BF	0.47	0.27–0.80	0.005	0.98	0.56–1.7	0.9
	Patient age per 10 years	1.09	0.99–1.20	0.08	1.05	0.93–1.19	0.4
	Secondary AML	1.45	1.11–1.90	0.007	1.36	1.07–1.72	0.011
	URD vs MSD	0.97	0.75–1.25	0.79	0.77	0.61–0.97	0.02
	Female donor to male recipient	0.79	0.60–1.03	0.078	1.04	0.78–1.37	0.8
	*In-vivo* TCD	0.96	0.74–1.23	0.7	0.99	0.72–1.37	0.97
	Dose Bu 12.8 vs 9.6 mg/kg	0.89	0.69–1.15	0.37			
	PBSC vs BM	1.01	0.76–1.3	0.95	0.84	0.56–1.28	0.42
	centre (frailty)			0.29			0.18
NRM	TBF vs BF	2.69	1.61–4.39	<10^–4^	0.79	0.39–1.58	0.5
	Patient age per 10 years	1.55	1.35–1.77	<10^–4^	1.27	1.06–1.52	0.01
	Secondary AML	0.89	0.61–1.3	0.55	1.39	1.01–1.9	0.04
	URD vs MSD	1.51	1.07–2.14	0.019	1.8	1.32–2.57	0.001
	Female donor to male recipient	1.12	0.82–1.53	0.47	1.49	1.05–2.12	0.025
	*In-vivo* TCD	0.86	0.61–1.21	0.38	0.72	0.47–1.09	0.12
	Dose Bu 12.8 vs 9.6 mg/kg	1.67	1.15–2.43	0.007			
	PBSC vs BM	1.73	1.14–2.65	0.01	0.79	0.45–1.40	0.42
	centre (frailty)			0.001			0.71
LFS	TBF vs BF	1.09	0.77–1.54	0.6	0.94	0.61–1.44	0.77
	Patient age per 10 years	1.22	1.13–1.33	<10^–4^	1.13	1.02–1.25	0.02
	Secondary AML	1.22	0.98–1.52	0.07	1.39	1.15–1.67	0.001
	URD vs MSD	1.13	0.92–1.40	0.24	1.02	0.85–1.23	0.85
	Female donor to male recipient	0.91	0.74–1.11	0.36	1.17	0.94–1.45	0.16
	*In-vivo* TCD	0.9	0.73–1.11	0.32	0.89	0.69–1.14	0.35
	Dose Bu 12.8 vs 9.6 mg/kg	1.15	0.93–1.42	0.21			
	PBSC vs BM	1.25	0.99–1.58	0.06	0.8	0.58–1.13	0.22
	centre (frailty)			0.13			0.88
OS	TBF vs BF	1.23	0.85–1.79	0.27	0.77	0.46–1.27	0.3
	Patient age per 10 years	1.28	1.18–1.4	<10^–4^	1.17	1.05–1.31	0.006
	Secondary AML	1.15	0.91–1.46	0.23	1.44	1.18–1.76	0.001
	URD vs MSD	1.01	0.88–1.37	0.4	1.25	1.02–1.53	0.03
	Female donor to male recipient	0.92	0.74–1.14	0.45	1.3	1.03–1.63	0.03
	*In-vivo* TCD	0.92	0.73–1.16	0.48	0.79	0.602–1.036	0.09
	Dose Bu 12.8 vs 9.6 mg/kg	1.09	0.87–1.38	0.45			
	PBSC vs BM	1.34	1.04–1.74	0.026	0.83	0.58–1.20	0.32
	centre (frailty)			0.057			0.89
GRFS	TBF vs BF	1	0.72–1.40	0.99	0.79	0.52–1.20	0.27
	Patient age per 10 years	1.17	1.09–1.26	<10^–4^	1.07	0.97–1.17	0.18
	Secondary AML	1.11	0.91–1.37	0.3	1.24	1.04–1.48	0.02
	URD vs MSD	1.06	0.88–1.29	0.56	1.11	0.94–1.32	0.23
	Female donor to male recipient	1.16	0.97–1.37	0.1	1.16	0.95–1.42	0.14
	*In-vivo* TCD	0.83	0.68–1.02	0.07	0.72	0.57–0.90	0.005
	Dose Bu 12.8 vs 9.6 mg/kg	1.06	0.86–1.30	0.6			
	PBSC vs BM	1.26	1.00–1.57	0.05	0.99	0.71–1.37	0.95
	centre (frailty)			0.005			0.19
aGVHD III–IV	TBF vs BF	1.34	0.71–2.53	0.37	0.69	0.23–2.04	0.5
	Patient age per 10 years	1.07	0.91–1.26	0.44	0.99	0.79–1.26	0.98
	Secondary AML	0.99	0.57–1.7	0.97	1.12	0.70–1.78	0.64
	URD vs MSD	1.08	0.68–1.71	0.75	3.09	1.87–5.10	<10^-4^
	Female donor to male recipient	1.03	0.65–1.6	0.89	1.46	0.87–2.44	0.15
	*In-vivo* TCD	0.89	0.57–1.37	0.57	0.54	0.29–1.08	0.05
	Dose Bu 12.8 vs 9.6 mg/kg	0.85	0.54–1.34	0.49			
	PBSC vs BM	1.89	1.05–3.42	0.03	2.05	0.64–6.61	0.23
	centre (frailty)			0.75			0.04
Severe cGVHD	TBF vs BF	1.13	0.6–2.12	0.71	0.46	0.15–1.35	0.16
	Patient age per 10 years	1.13	0.99–1.30	0.08	0.95	0.8–1.14	0.6
	Secondary AML	1.01	0.65–1.57	0.98	0.94	0.62–1.42	0.78
	URD vs MSD	1.3	0.87–1.95	0.20	1.18	0.82–1.71	0.38
	Female donor to male recipient	1.91	1.42–2.58	<10^–4^	1.26	0.83–1.89	0.27
	*In-vivo* TCD	0.33	0.21–0.51	<10^–4^	0.48	0.30–0.78	0.003
	Dose Bu 12.8 vs 9.6 mg/kg	1.15	0.75–1.76	0.52			
	PBSC vs BM	1.54	0.98–2.42	0.06	1.2	0.57–2.54	0.63
	centre (frailty)			0.002			0.02

Abbreviations: aGVHD, acute graft-versus-host disease; BF, busulfan-fludarabine; BM, Bone marrow; Bu, busulfan; cGVHD, chronic graft-versus-host disease; GRFS, graft-versus-host-free, relapse-free survival; MAC, myeloablative conditioning; LFS, leukemia-free survival; MSD, matched sibling donor; NRM, non-relapse mortality OS, overall survival; URD, unrelated donor; PBSCs, peripheral blood stem cells; RIC, reduced-intensity conditioning; TBF, thiotepa-busulfan-fludarabine; TCD, T-cell depletion.

### Transplant outcome

**Figure 1:**
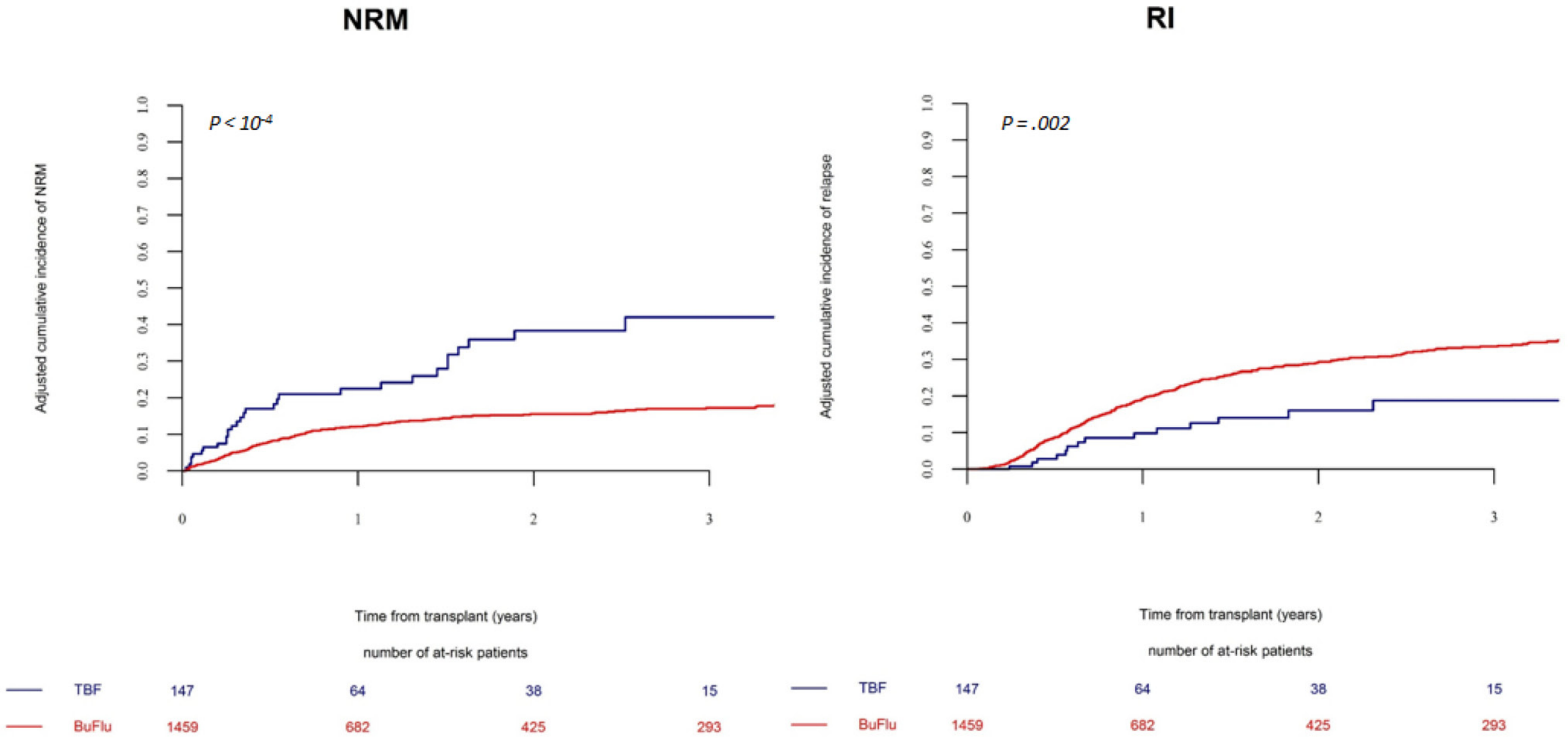
Non-relapse mortality and relapse incidence in patients receiving a myeloablative regimen The cumulative incidence of non-relapse mortality and relapse by conditioning regimen (TBF-MAC vs BF-MAC).

The 2-year relapse incidence was significantly lower in TBF-MAC group (14 ± 6%) compared to BF-MAC group (27 ± 2%, *p =* 0.002) (Figure 1). This result was strongly confirmed in multivariate analysis (HR 0.5, *p =* 0.005). Secondary AML was the only additional factor associated with higher relapse risk in multivariate analysis (Table 3).

The reduced relapse following TBF-MAC was particularly evident in the early post transplant period; in fact, relapse incidence before day 100 was 2% (95% CI: 1–6) for TBF-MAC vs 9% (95% CI: 7–10) for BF-MAC (*p =* 0.007), while no significant difference in relapse incidence was observed after day 100 (13% (95% CI: 7–20) vs 17% (95% CI: 14–19) for TBF-MAC and BF-MAC respectively, *p =* 0.4).

**Figure 2:**
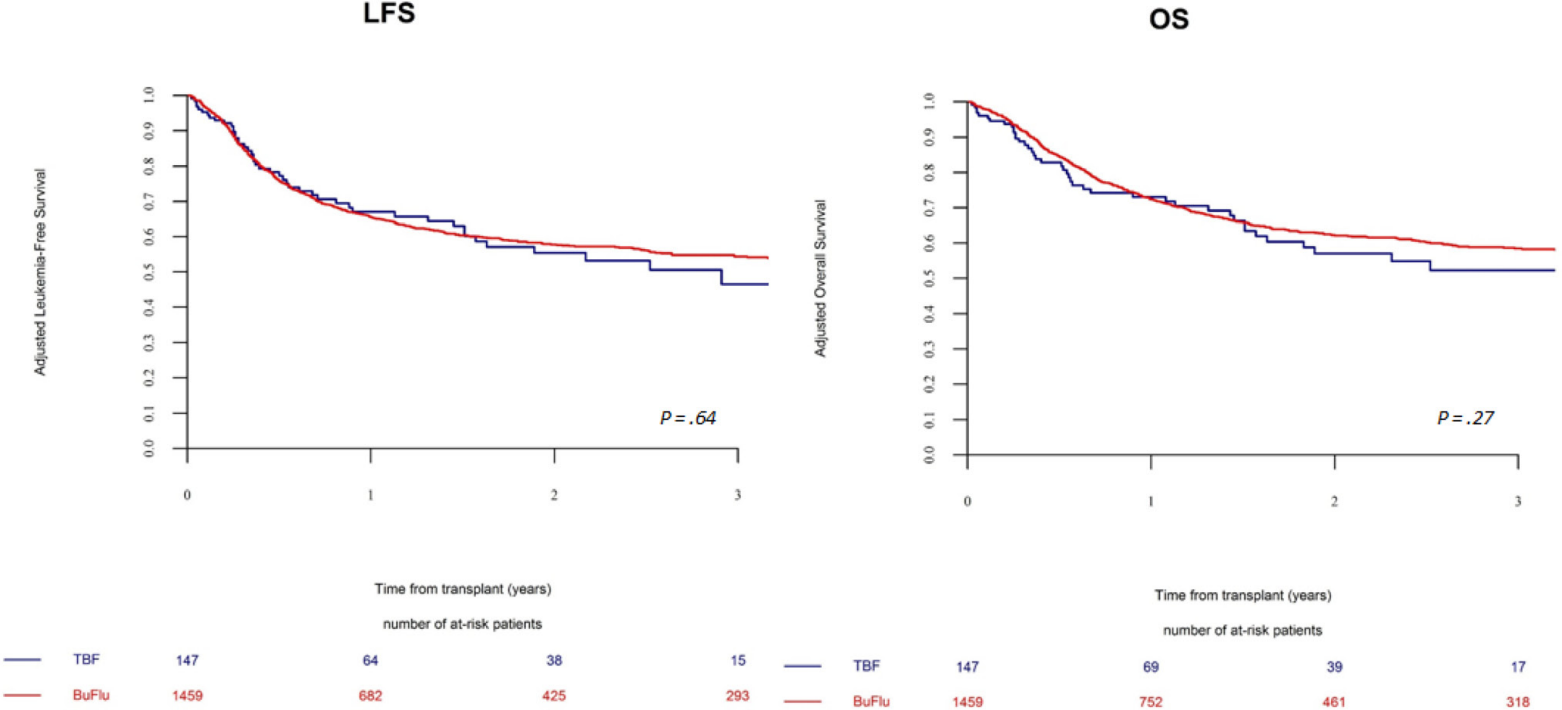
Leukemia free and overall survival in patients receiving a myeloablative regimen The probabilities of leukemia-free and overall survival by conditioning regimen (TBF-MAC vs BF-MAC).

Leukemia-free survival (LFS) at 2 years was not statistically different between TBF-MAC and BF-MAC groups, being 59 ± 10% in TBF-MAC and 57 ± 3% in BF-MAC, respectively (*p =* 0.5) (Figure 2). Multivariate analysis confirmed those results. The only predictive factor independently associated with LFS in multivariate analysis was age at transplant. Overall survival at 2 years also did not differ between the two regimens, being 62 ± 10% and 61 ± 3% in TBF-MAC and BF-MAC, respectively (*p =* 0.87). This result was confirmed by multivariate analysis. Factors which negatively affected survival in multivariate analysis were older age at transplant and PBSCs as stem cell source. The composite endpoint GVHD-free, relapse-free survival (GRFS) rate at 2 years did not differ between the 2 conditioning regimens being 52% in TBF-MAC and 41% in BF-MAC, respectively (*p =* 0.2). Importantly, the use of ATG was independently associated with significantly lower probability of severe cGVHD and with a trend for better GRFS. In the population of patients who relapsed after transplant, there was no difference in overall survival after relapse between the two study cohorts. In MAC group 2-year after relapse OS was 14% vs 10% in the TBF and BF groups, respectively (*p =* 0.74). In RIC cohort OS was 17% vs 13% one year after relapse (*p =* 0.86).

### Subgroup analysis

**Figure 3:**
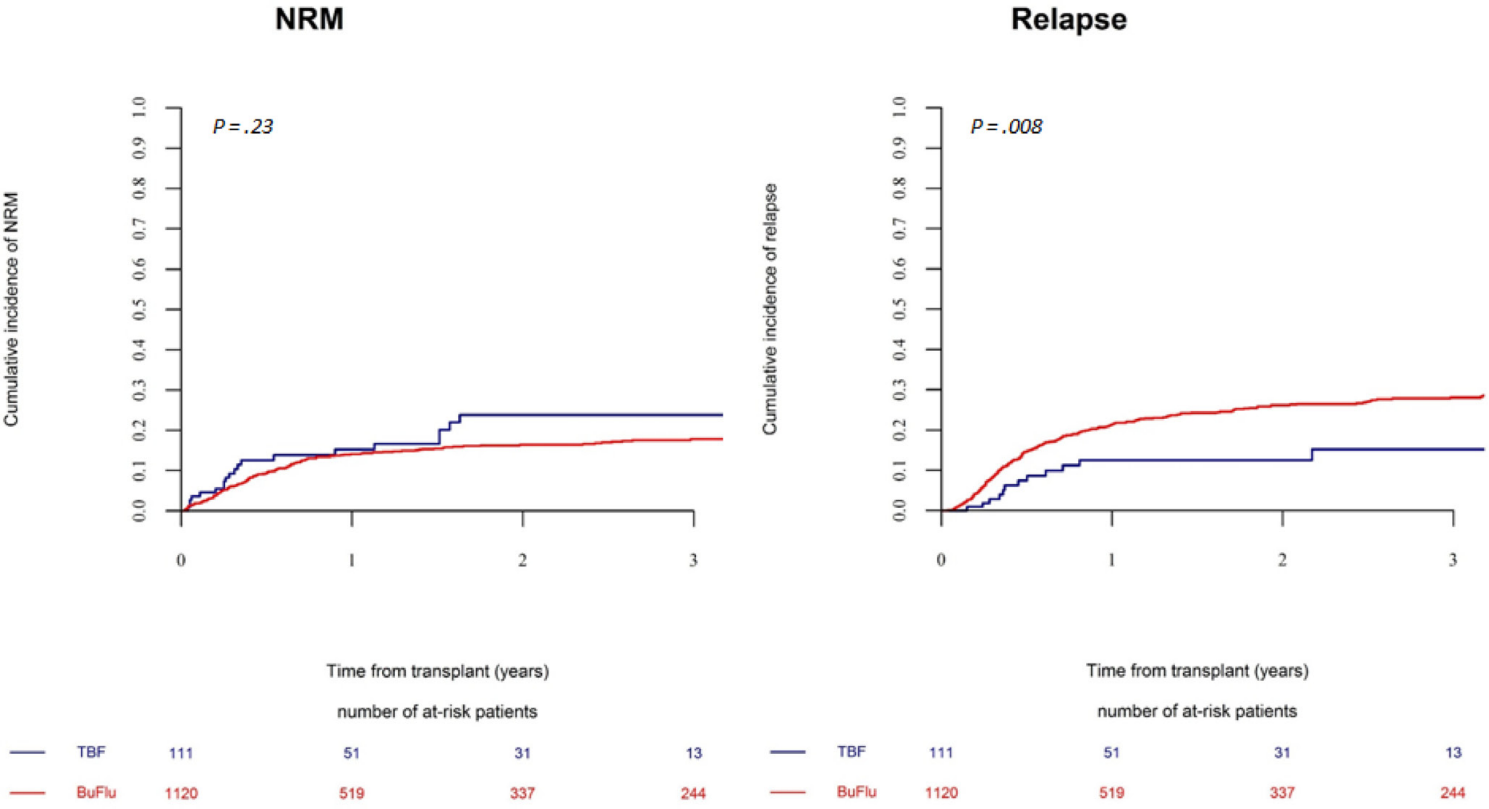
Non-relapse mortality and relapse incidence in patients receiving a myeloablative regimen with selected busulfan doses The cumulative incidence of relapse and non-relapse mortality by conditioning regimen (TBF with busulfan 9.6 mg/kg vs BF with busulfan 12.8 mg/kg).

In multivariate analysis conducted in the global population, busulfan dose (12.8 vs 9.6 mg/kg) emerged as independently associated with NRM, while had no impact on relapse risk. Given this findings, and according to the regimens schedules as previously published, [10, 17] we conducted a subgroup analysis of TBF-MAC patients who received busulfan 9.6 mg/kg, which were compared to BF-MAC patients receiving busulfan 12.8 mg/kg (Table 4). With the reduced dose of busulfan (9.6 mg/kg) the NRM risk following TBF-MAC resulted still higher however not significantly different as compared to BF-MAC (HR 1.53, *p =* 0.12). Notably, despite the reduced busulfan dosage, relapse risk following TBF-MAC remained significantly lower as compared to BF-MAC (HR 0.45, *p =* 0.01) (Figure 3). Finally, no impact of the kind of conditioning regimen could be observed on LFS, OS or GRFS (TBF-MAC vs BF-MAC: LFS: HR 0.82, *p =* 0.31; OS: HR 0.95, *p =* 0.82; GRFS: HR 0.78, *p =* 0.18). Results of multivariate analysis in this subgroup are presented in Table 4.

**Table 4:** Multivariate analysis of transplantation outcome in TBF-MAC vs BF-MAC with selected busulfan dose

Outcome		HR	95% CI	*P*
Relapse	TBF vs BF	0.45	0.25–0.83	0.01
	Patient age per 10 years	1.13	1.02–1.25	0.02
	Secondary AML	1.25	0.88–1.77	0.2
	URD vs MSD	1.11	0.81–1.52	0.49
	Female donor to male recipient	0.96	0.72–1.29	0.8
	*In-vivo* TCD	0.82	0.62–1.09	0.19
	PBSC vs BM	0.96	0.72–1.29	0.81
	centre (frailty)			0.69
NRM	TBF vs BF	1.53	0.89–2.6	0.12
	Patient age per 10 years	1.57	1.35–1.82	<10^–5^
	Secondary AML	0.88	0.56–1.38	0.58
	URD vs MSD	1.61	1.08–2.38	0.02
	Female donor to male recipient	1.12	0.79–1.58	0.53
	*In-vivo* TCD	0.814	0.562–1.177	0.27
	PBSC vs BM	1.94	1.21–3.12	0.006
	centre (frailty)			0.004
LFS	TBF vs BF	0.82	0.56–1.2	0.31
	Patient age per 10 years	1.26	1.16–1.38	<10^–5^
	Secondary AML	1.08	0.82–1.42	0.59
	URD vs MSD	1.27	0.99–1.61	0.059
	Female donor to male recipient	1.03	0.82–1.29	0.79
	*In-vivo* TCD	0.8	0.63–1.01	0.05
	PBSC vs BM	1.27	0.98–1.64	0.07
	centre (frailty)			0.22
OS	TBF vs BF	0.95	0.63–1.44	0.82
	Patient age per 10 years	1.32	1.19–1.45	<10^–5^
	Secondary AML	0.99	0.73–1.33	0.92
	URD vs MSD	1.21	0.92–1.58	0.17
	Female donor to male recipient	1.02	0.81–1.3	0.85
	*In-vivo* TCD	0.82	0.64–1.06	0.13
	PBSC vs BM	1.37	1.03–1.82	0.03
	centre (frailty)			0.14
GRFS	TBF vs BF	0.78	0.54–1.126	0.18
	Patient age per 10 years	1.21	1.118–1.319	<10^–5^
	Secondary AML	1.04	0.807–1.344	0.76
	URD vs MSD	1.21	0.963–1.52	0.10
	Female donor to male recipient	1.25	1.027–1.517	0.76
	*In-vivo* TCD	0.75	0.595–0.933	0.01
	PBSC vs BM	1.29	1.007–1.663	0.04
	centre (frailty)			0.02

### Propensity score matched-pairs analysis

Primary and secondary endpoints were further challenged in a propensity score matched-pairs analysis, conducted on 138 TBF-MAC matched with 262 BF-MAC patients, which confirmed the results obtained in the global population (Supplementary Tables 1 and 2, appendix).

### Reduced-intensity conditioning: TBF-RIC versus BF-RIC

One thousand three hundred and four patients received a RIC regimen; among them, 65 patients conditioned with TBF-RIC were compared to 1239 patients who received BF-RIC. Characteristics of patients are summarized in Table 1.

Engraftment rate was 96% following TBF-RIC and 99% after BF-RIC, respectively (*p =* 0.063). The median time to neutrophil engraftment was 16 (8–29) days and 18 (8–97) days for TBF-RIC and BF-RIC, respectively (*p =* 0.01). The incidence of grade II–IV and III–IV aGVHD was similar between the groups, being 22 ± 9% and 6 ± 4% in the TBF-RIC and 22 ± 3% and 8 ± 2% in the BF-RIC group (*p =* 0.79 and *p =* 0.59), respectively.

**Figure 4:**
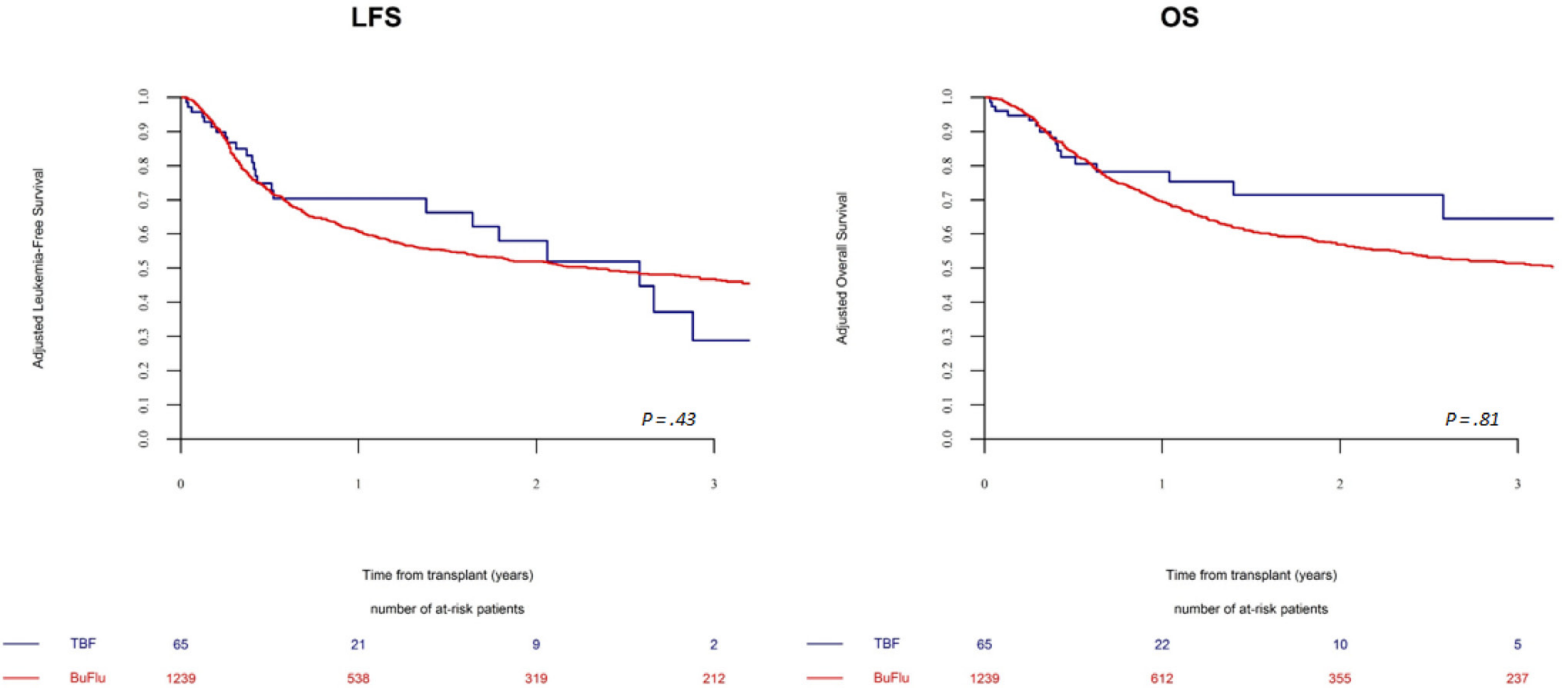
Leukemia free and overall survival in patients receiving a reduced intensity regimen The probabilities of leukemia-free and overall survival by conditioning regimen (TBF-RIC vs BF-RIC).

Leukemia-free survival and overall survival did not differ between TBF-RIC and BF-RIC, being 47 ± 18% vs 52 ± 3% (*p =* 0.43) and 60 ± 17% vs 57 ± 3% (*p =* 0.81), respectively (Figure 4).

Multivariate analysis confirmed those results (Table 3). The 2-year NRM and relapse rates were not statistically different between the two study groups, being 15 ± 8% vs 18 ± 2% (*p =* 0.62) and 39 ± 18% vs 30 ± 3% (*p =* 0.62), respectively. The 2-year cumulative incidence of cGVHD was similar between the groups, and so was the composite endpoint GRFS.

The use of ATG was independently associated with significantly lower probability of severe cGVHD and better GRFS.

Similarly to the MAC cohort, endpoints were further tested by a propensity score matched-pairs analysis, conducted on 61 TBF-RIC vs 118 BF-RIC patients, which confirmed these results (Supplementary Tables 1 and 2, appendix).

## DISCUSSION

Despite great efforts in optimizing preparatory regimens and transplant procedures, leukemia relapse remains today the major cause of transplant failure. We hypothesized that the thiotepa-busulfan-fludarabine protocol could ensure a strong leukemia control, reducing relapse and thus leading to improved outcome as compared to busulfan-fludarabine.

In fact, the TBF-MAC conditioning was associated with inferior relapse as compared to BF-MAC; however, the increased non-relapse mortality offset this advantage and no difference could be observed in survival between the two regimens. Notably, the 2-year relapse incidence was as low as 14% following the TBF-MAC; in multivariate analysis, relapse risk was cut by half using the TBF-MAC as compared to the BF-MAC protocol. These results compare favourably with myeloablative regimens currently employed; for instance, 2-year relapse rate following the classical CY/TBI regimen stands at about 20% when transplant is given in remission [25]. In fact, leukemia recurrence following transplant remains an unresolved issue, and the recent tendency to mitigate conditioning regimen intensity raises significant concerns about further increasing relapse risk, as confirmed by recent evidence [26]. In their randomized BF vs BuCy study, Lee and colleagues [8] reported a 2-year relapse-free survival of 55% following BF, which was significantly inferior as compared to BuCy; Rambaldi *et al.* [10] observed a cumulative incidence of relapse following BF of about 30% at 2 years which however, in contrast to the previously cited study, was not statistically different as compared to BuCy.

The addition of thiotepa to busulfan and fludarabine appears to provide a significantly stronger anti leukemic effect; in fact, our results concur with recent observations reporting encouraging low relapse rates when TBF was employed in cord blood (Sanz *et al.*, [17] 5-year RI: 18%) and haplo-SCT (Bacigalupo *et al.*, [19] cumulative incidence of relapse related death: 11% for patients in CR1). In the study by Di Bartolomeo *et al.*, [21] the use of TBF-MAC was the only factor predicting lower relapse risk in multivariate analysis. It might be speculated that the combined efficacy of two alkylating agents mediating powerful anti-leukemic activity could be responsible of such effective disease control. In addition, thiotepa is well known to have a radiomimetic action and to penetrate sanctuary sites [27].

Nevertheless, the low relapse rate following TBF observed in our study should be interpreted with caution as it could, at least in part, be related to the high NRM observed in the same cohort of patients. Indeed, 2-year NRM following TBF-MAC was 27% in the overall population, which resulted significantly higher as compared to the BF conditioning. Higher NRM following a strong myeloablative protocol (TBF) as compared to a reduced-toxicity regimen (BF) does not represent a surprising finding per se; further, this result is in accordance with the previously cited studies in which TBF-MAC was employed in haploidentical (Arcese *et al.* [22] cumulative incidence of NRM: 32%) and cord blood transplant (Sanz *et al.*, [17] 5-year NRM: 44%). However, the significant NRM rate reported in our study warrants a deeper reflection. When examining factors associated with NRM, busulfan dose (12.8 vs 9.6 mg/kg) stood out as strongly predicting high NRM risk, while it did not demonstrate any impact on relapse incidence. In view of this observation, and considering the published standard regimen schedules, [10, 17] we performed a subgroup analysis comparing TBF-MAC with busulfan 9.6 mg/kg and BF-MAC with busulfan 12.8 mg/kg. With the reduced dose of busulfan, NRM risk after TBF substantially decreased to a level not statistically different as compared to BF; however a trend towards higher NRM for TBF was retained. Importantly, the lower dose of busulfan did not impair the anti-leukemic activity of TBF, as relapse remained significantly inferior as compared to BF. Based on these data, the combination of two alkylators at full myeloablative doses appears excessively toxic, with no apparent benefit in terms of disease control. Nevertheless in this subgroup analysis, similarly to what was found in the global population, the strong advantage of TBF-MAC in terms of reduced relapse was negated by the high (although slightly reduced) NRM. Indeed, despite TBF hazard ratios for LFS, OS and GRFS resulted constantly below 1, no statistical difference in survival could be observed between the two regimens.

When conducting exploratory analyses according to age, donor type and leukemia risk, we could not find any significant difference between TBF-MAC and BF-MAC in terms of survival in any of the subgroups examined.

The main causes of death were infection and GVHD. Lethal infectious complications accounted for one out of four deaths in the TBF group. Our results are in accordance with previous publications reporting high rate of infection following TBF regimen [22]. Thiotepa holds a heavy myeloablative effect combined with a powerful immunosuppressive potential which may have contributed to this. The acknowledgment of such risks following TBF-MAC could help to better select patients suitable for this regimen, and to implement an optimal anti-infectious monitoring, prophylaxis and treatment which may improve outcome.

Notably, despite the higher intensity, TBF-MAC resulted in similar incidence of grade II-IV aGVHD as compared to BF-MAC. The strong immunosuppressive activity of thiotepa in association with fludarabine may have played a role in this. Finally, incidence of severe cGVHD was similar between the two conditioning regimens. Importantly, the inclusion of ATG in both protocols was independently associated with significantly lower probability of severe cGVHD both in patients receiving MAC and RIC, and with better GRFS.

In a separate analysis, we compared reduced-intensity versions of TBF and BF, reporting similar outcome. Interestingly, NRM rate of TBF-RIC was similar to BF-RIC, despite the combination of two alkylators in a cohort of patients with a median age of 60 years. We did not observe a difference in relapse incidence between the two study cohorts; however, due to the limited number of patients receiving TBF-RIC no definite conclusion can be made.

It is important to recognize the limitations of the present study. First, the retrospective design did not allow to study the reason for patient allocation to a specific regimen. Secondly, the two study arms were unbalanced as to the number of patients included. Finally, some of the patient’s characteristics varied among the two groups. Nonetheless, the present analysis represents the largest study reporting outcome of TBF conditioning in MSD or URD-SCT for AML; further, we addressed the inherent limitations of a registry-based study performing a confirmatory PS matched-pair analysis, which thoroughly upheld all the results we observed in the overall population.

## CONCLUSIONS

In conclusion, our results suggest that TBF-MAC provides significantly lower relapse, which is counterbalanced by increased NRM as compared to BF-MAC, thus resulting in similar survival. The combination of thiotepa with high dose busulfan (12.8 mg/kg) appears excessively toxic, while TBF with lower dose of busulfan (9.6 mg/kg) seems to retain strong anti-leukemic effect in combination with acceptable NRM, however with no statistically significant survival advantage over BF. In elderly patients, TBF-RIC appears a promising reduced-intensity regimen, as similar outcome was reported as compared to BF-RIC. This registry data should be validated by a well-designed randomized trial comparing outcome of TBF with other busulfan-based regimens, both in the MAC and RIC setting. Specifically, designed studies are necessary to identify which patient category could benefit the most from the strong anti-leukemic potential of TBF protocol, without an excess of NRM. Young, fit patients at very high risk of relapse might candidate for TBF regimen, providing a careful patient selection and an optimal supportive care in order to minimize transplant toxicity.

## METHODS

### Study design and data collection

This is a registry based retrospective study. Data were provided and the study design was approved by the acute leukemia working party (ALWP) of the EBMT group registry, in accordance with the EBMT guidelines for retrospective studies. EBMT is a voluntary working group of more than 500 transplant centers which are required to report all consecutive stem cell transplantations and follow-up once a year. Audits are routinely performed to determine the accuracy of the data. Since 1990, patients have been able to provide informed consent that authorizes the use of their transplant information for research purposes. The ALWP of the EBMT granted ethical approval for this study. We included in the analysis patients with AML older than 18 at diagnosis, who had received either TBF or BF as conditioning regimen for MSD or URD SCT in CR1 as first transplant between January 2007 and June 2015, reported to the EBMT. All unrelated donors were HLA-matched (10/10) or mismatched at one HLA locus (9/10). Patients who received conditioning regimens including oral busulfan, T-depleted grafts, or transplant from <9/10 mismatched unrelated donor were excluded. Myeloablative conditioning regimen (MAC) was defined as ivBusulfan dose ≥9.6 mg/kg (TBF-MAC and BF-MAC), while reduced-intensity conditioning (RIC) was defined as iv Busulfan dose of 6.4 mg/kg (TBF-RIC and BF-RIC) in accordance with the EBMT definitions [28].

### End-point definitions and statistical analysis

Primary end-points were overall survival (OS) and leukemia-free survival (LFS). Secondary end-points were relapse incidence (RI), non-relapse mortality (NRM), graft-versus-host free, relapse-free survival (GRFS), engraftment, incidence and severity of acute (aGVHD) and chronic graft-versus-host disease (cGVHD). The severity of acute GVHD was graded on a I–IV scale, while cGVHD was scored as mild, moderate or severe in accordance to EBMT standards [28]. LFS was defined as the interval from transplant to either relapse or death. OS was defined as the time between the date of transplant and the date of death. GRFS was defined as alive with no previous grade III–IV aGvHD, no severe chronic GvHD and no relapse [29]. Probabilities of OS, LFS and GRFS were estimated using Kaplan-Meier curves. Cumulative incidence functions were used to estimate relapse incidence (RI) and non-relapse mortality (NRM) in a competing risks setting. In order to study acute and chronic GVHD, we considered death and relapse as competing events. The main patient characteristics were compared using Mann-Whitney test for quantitative variables, chi-square test or Fisher exact test for categorical variables. Univariate analyses were performed using the log rank test for OS, LFS and GRFS, the Gray test for cumulative incidences. Multivariate analyses were performed using the Cox proportional-hazard model. Factors differing between two groups in terms of distribution and factors significantly associated with the outcome were included in the multivariate analysis. In order to test for a centre effect, we introduced a random effect of frailty for each centre into the model [30]. Finally, a propensity score matched pairs analysis was conducted to corroborate the results obtained in the global population; detailed statistics and results are presented in the appendix. All tests were two-sided and *P* values < 0.05 were considered as indicating a statistically significant association. Analyses were performed using the R statistical software version 3.2.3 (available online at http://www.R-project.org), and propensity score analysis was performed using the ‘MatchIt’ [31].

## SUPPLEMENTARY MATERIALS TABLES





## Abbreviations

aGVHD: acute graft-versus-host disease
BF: busulfan-fludarabine
BM: Bone marrow
Bu: busulfan
cGVHD: chronic graft-versus-host disease
GRFS: graft-versus-host-free, relapse-free survival
MAC: myeloablative conditioning
LFS: leukemia-free survival
MSD: matched sibling donor
NRM: non-relapse mortality
OS: overall survival
URD: unrelated donor
PBSCs: peripheral blood stem cells
RIC: reduced-intensity conditioning
TBF: thiotepa-busulfan-fludarabine
TCD: T-cell depletion
